# Prognostic significance of neutrophil-to-lymphocyte ratio in prostate cancer: evidence from 16,266 patients

**DOI:** 10.1038/srep22089

**Published:** 2016-02-25

**Authors:** Xiaobin Gu, Xianshu Gao, Xiaoying Li, Xin Qi, Mingwei Ma, Shangbin Qin, Hao Yu, Shaoqian Sun, Dong Zhou, Wen Wang

**Affiliations:** 1 Department of Radiation Oncology, Peking University First Hospital, Beijing 100034, China

## Abstract

This study was aimed to investigate the prognostic value of neutrophil-to-lymphocyte ratio (NLR) in patients with prostate cancer (PCa). A meta-analysis including 14 publications (15 cohorts) with 16,266 patients was performed to evaluate the association between NLR and overall survival (OS), progression-free survival (PFS)/recurrence-free survival (RFS) in PCa using hazard ratio (HR) and 95% confidence intervals (95% CI). The combining data showed that increased NLR predict poor OS (HR = 1.38, 95%CI: 1.22–1.56) and PFS/RFS (HR = 1.24, 95%CI 1.05–1.46) in PCa. Stratified analysis by PCa type, sample size, ethnicity and NLR cut-off value revealed that NLR showed consistent prognostic value in metastatic castration-resistant prostate cancer (mCRPC) patients and predict poor PFS/RFS in Asians, but not in Caucasians. These statistical data suggested that increased NLR could predict poor prognosis in patients with PCa.

Prostate cancer (PCa) is the most frequently diagnosed cancer and the second leading cause of cancer-related death among men in the western world, accounting for 220,800 new cases and 27,540 deaths in the US annually^1^. The incidence of prostate cancer varies remarkably in different countries and regions, which increased significantly during the past few decades due to diverse approaches diagnosing PCa. Despite different treatment methods including radical prostatectomy, hormone deprivation therapy, radiation therapy and chemotherapy were applied, most tumors relapse in 2 years to the castration-resistant state and the prognosis of PCa remains disappointing^2^. Given this, it is of interest for clinicians to stratify the high risk PCa patients who are prone to tumor recurrence and poor prognosis so that clinicians could further identify optimal treatment strategies^3^.

Accumulating evidence has shown that inflammation response is closely associated with tumorigenesis and tumor progression^4^,^5^. The interactions between tumor and inflammation were complex and involved different mechanisms. Inflammation plays an important role in every single step in carcinogenesis, involving tumor initiation, angiogenesis promotion, apoptosis inhibition and tumor metastasis^6^. The changes in systemic inflammatory response can be reflected by measurement of various blood-based parameters. A variety of blood-based indexes including C-reactive protein (CRP), modified Glasgow Prognostic Score (mGPS), platelets count and neutrophil-to-lymphocyte ratio (NLR) have been extensively explored to predict prognosis of cancer patients^7^,^8^,^9^. A large amount of studies have reported that elevation of NLR was associated with poor clinical outcomes in various malignant tumors^10^,^11^. In recent years, several studies have reported that an elevation in NLR correlated with biological tumor recurrence and poor prognosis in patients with PCa^12^,^13^,^14^. However, some other studies did not detect the prognostic significance of NLR in PCa patients^15^,^16^. Therefore, it is necessary to systematically clarify the prognostic significance of NLR in PCa by the approach of meta-analysis.

The current study was designed to evaluate the prognostic value of elevated NLR for overall survival (OS) and progress-free survival (PFS)/recurrence-free survival (RFS) in patients with PCa by pooling results from published data.

## Results

### Selection and characteristics of included eligible studies

**Figure 1:**
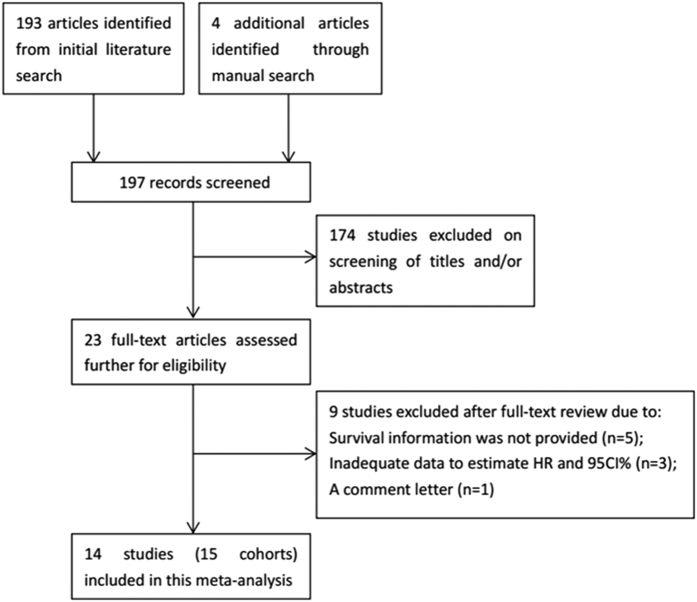
The flow chart of literature selection.

The selection process was shown in Fig. 1. Through initial searching of the database, 197 records were retrieved and screened by title and abstract. 174 records were discarded after title and abstracts screening because they were duplicate records, reviews, irrelevant studies and nonhuman studies. 23 full-text records were further evaluated for eligibility. Of them, nine studies were eliminated due to following reasons: five did not provide the survival information, three provided insufficient data to calculate HR and 95%CI, one was a comment. Soest *et al.*^17^ included two independent randomized phase III trials in their paper, we marked the cohorts as Soest1 and Soest2. Finally, 14 publications^12^,^13^,^14^,^15^,^16^,^17^,^18^,^19^,^20^,^21^,^22^,^23^,^24^,^25^ (15 cohorts) with 16,266 patients published between 2012 and 2015 were included in the meta-analysis process. The basic information of the included studies was summarized in Table 1. 11 studies (12 cohorts)^12^,^13^,^14^,^15^,^16^,^17^,^19^,^20^,^21^,^23^,^24^ were from western countries, 3 studies^18^,^22^,^25^ were from Asian countries. Of the 14 studies (15 cohorts), 10 studies (11 cohorts)^12^,^13^,^14^,^15^,^16^,^17^,^18^,^19^,^21^,^24^ investigated the prognostic role of NLR on OS, 9 studies^14^,^18^,^19^,^20^,^21^,^22^,^23^,^24^,^25^ investigated prognostic role of NLR on PFS/RFS.

### NLR and OS in PCa

**Figure 2:**
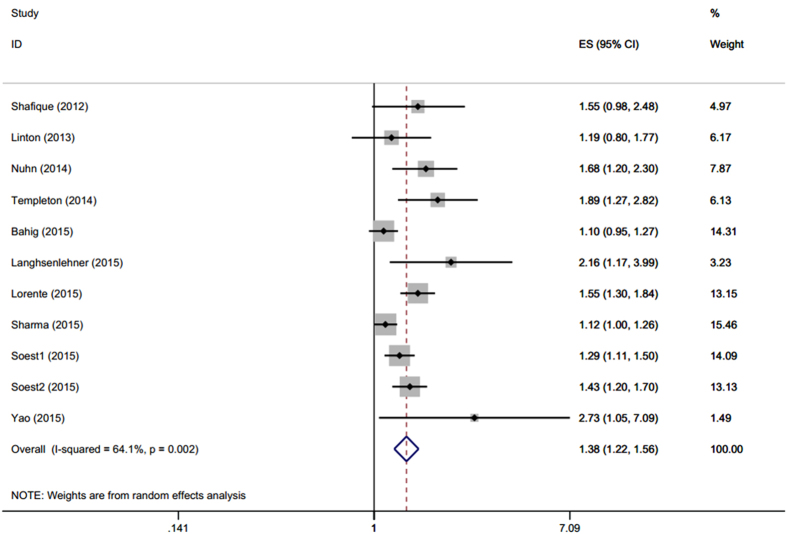
Meta-analysis of the association between elevated NLR and OS.

11 cohorts with 14,250 patients provided the data of NLR and OS in PCa. The pooled HR of 1.38 (95%CI: 1.22–1.56) with heterogeneity (P_h_ = 0.002, I^2^ = 64.1%) showed elevated NLR predicted shorter OS in patients with PCa (Fig. 2, Table 2).

### NLR and PFS/RFS in PCa

**Figure 3:**
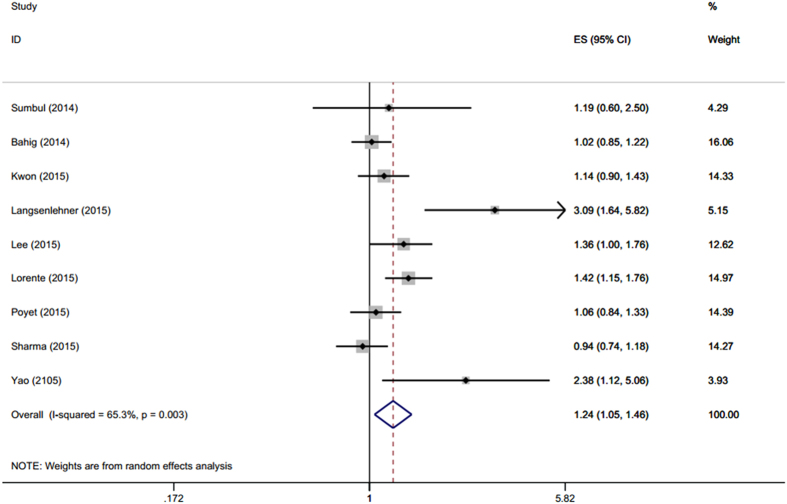
Meta-analysis of the association between elevated NLR and PFS/RFS.

9 cohorts with 12541 patients presented the data of pretreatment NLR and PFS/RFS in PCa. Though with heterogeneity (P_h_ = 0.003, I^2^ = 65.3%), a significant correlation between increased NLR and poor PFS/RFS (HR = 1.24, 95%CI: 1.05–1.46) was detected according to our pooled data (Fig. 3, Table 2). Interestingly, increased NLR predicted shorter RFS/PFS in Asian populations (HR = 1.42, 95%CI: 1.11–1.82), but not in Caucasian populations (HR = 1.18, 95%CI: 0.98–1.43).

### Subgroup analysis and meta-regression

Subgroup analysis and meta-regression were carried out to investigate the sources of heterogeneity. With respect to the correlation between NLR and OS, subgroup analysis stratified by PCa types showed that the combined HRs were 1.44 (95%CI: 1.32–1.57) for metastatic castration-resistant prostate cancer (mCRPC) and 1.16 (95%CI: 0.98–1.36) for localized PCa. Subgroup analysis dichotomized by sample size(>400 vs. <400) and NLR cut-off value (≤3 vs. >3) did not change the results substantially(Table 2). Meta-regression showed PCa types could be the potential source of heterogeneity (p = 0.01). With regard to the correlation between NLR and PFS/RFS, subgroup analysis showed similar results with total results. Notably, the pooled HR for Asian ethnicity was 1.42 (95%CI: 1.11–1.82) without significant heterogeneity (P_h_ = 0.345, I^2^ = 6%). In addition, the pooled HR for mCRPC was 1.45 (95%CI: 1.19–1.77) with good homogeneity (P_h_ = 0.37, I^2^ = 0) (Table 2).

### Sensitivity analysis

**Figure 4:**
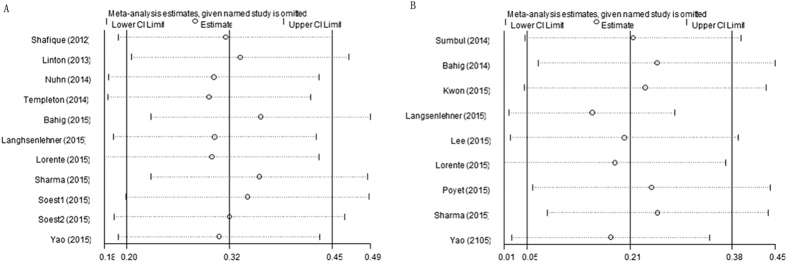
Sensitivity analysis on the relationship between NLR and (**A**) OS and (**B**) PFS/RFS in PCa.

Omitting any single study by turn, sensitivity analysis demonstrated that the combined HRs for OS and PFS/RFS did not significantly alter (Fig. 4).

### Publication bias

**Figure 5:**
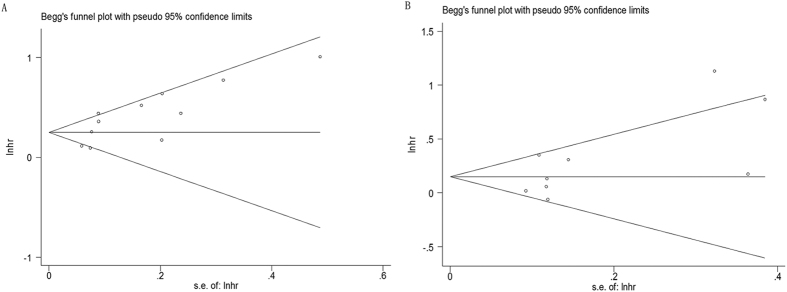
Begg’s funnel plot of publication bias test for (**A**) OS and (**B**) PFS/RFS in PCa.

Evaluation of publication bias using Begg’s test (p < 0.05 was considered as statistical significant) demonstrated that there was no significant publication bias in OS and PFS/RFS studies (p = 0.119 and p = 0.251, respectively)(Fig. 5).

## Discussion

In the present study, we aimed to explore the prognostic value of NLR in patients with PCa. A total of 14 studies (15 cohorts) containing 16,266 patients were included in this meta-analysis to calculate pooled HR. The results showed that increased pretreatment NLR was associated with poor OS(HR = 1.38, 95%CI: 1.22–1.56) and PFS/RFS (HR = 1.24, 95%CI: 1.05–1.46), though with heterogeneity. Subgroup analysis divided by ethnicity, PCa type, sample sizes and NLR cut-off value did not significantly change the main results. Of note, subgroup analysis demonstrated that pretreatment NLR had enhanced prognostic efficiency for OS and PFS/RFS in mCRPC (HR = 1.44, 95%CI: 1.32–1.57 for OS and HR = 1.45,95%CI: 1.19–1.77 for RFS/PFS) without significant heterogeneity. In addition, elevated NLR also predicted poor PFS/RFS in Asian populations, but not in Caucasian population. To our knowledge, our study is the first meta-analysis exploring the prognostic effects of increased NLR in OS and PFS/RFS in patients with PCa.

Growing evidence has indicated that inflammatory response could be heavily involved in the occurrence and development of different cancer types^26^,^27^,^28^. Studies revealed that inflammation-related neutrophils and immunocytes including lymphocytes were indispensable participants in tumorigenesis^29^. Inflammation exerts an important role in tumor formation and development through facilitating angiogenesis, proliferation and protecting tumors from apoptosis. By secreting a variety of chemokines, tumor cells could attract pro-inflammatory cells into tumor microenvironment, subsequently, an array of cytokines produced by neutrophils stimulate tumor cells growth^30^. Of these inflammatory parameters reflecting the systemic inflammatory response, an increased neutrophil-to-lymphocyte ratio (NLR) has been found valuable to predict clinical outcome of cancer patients^31^. Additionally, NLR is obtained from routine blood test, making it an easily available and reliable marker. A large amount of studies have showed the correlation between increased pretreatment NLR and poor prognosis in different malignant tumors including gastric cancer, colorectal cancer, breast cancer, prostate cancer, soft-tissue sarcoma and non-small cell lung cancer^17^,^27^,^32^,^33^,^34^,^35^.

Our results demonstrated that elevated NLR was in associated poor OS and PFS/RFS in patients with PCa, which was in accordance with the results from meta-analysis with other cancer types^34^,^36^,^37^. We have noted that a recently published work investigated the prognostic value of NLR in various solid tumors^11^. However, in that meta-analysis^11^, only three studies concerning patients with castration resistant prostate cancer were included. In the present work, we included patients with both localized PCa and mCRPC in an adequately sufficient data. Moreover, subgroup analysis was performed, which could provide detailed information for clinical management. More importantly, we found that NLR has adequate prognostic value for OS and PFS/RFS in mCRPC. Although chemotherapy and hormone therapy were used, mCRPC commonly occurred after the treatment after few years. Therefore, the NLR measurement could provide valuable prognostic information for mCRPC and be helpful for optimal treatment strategies selection. In addition, our results showed increased NLR predicted poor PFS/RFS in Asians, but not in Caucasians, which could be attributed to the ethnicity heterogeneity. Notably, corticosteroids were one of the palliative treatment options in patients with mCRPC for 30 years^38^. Intake of corticosteroids had immunosuppressive effects, which could influence the value of NLR^21^. The prognostic value of NLR in PCa should be evaluated after adjustment on potential confounders including use of corticosteroids. However, only one^21^ of the included studies provided the relevant data, thus the analysis could not be conducted in the current meta-analysis due to insufficient data. Interestingly, Lorente *et al.*^21^ found that NLR had independent prognostic value on OS regardless of corticosteroids usage using multivariable analysis. However, a recent work^39^ found that, in patients with melanoma, elevated NLR and corticosteroids before week 1 were associated with poorer OS in univariate analysis, however, in multivariate analysis, elevated NLR remained an independent prognostic factor whereas the prognostic efficiency of intake of corticosteroids disappeared. Since corticosteroids were widely used drugs in mCRPC treatment, further studies should take into account intake of corticosteroids when performing survival analysis. Furthermore, more large scale studies are needed to explore the effects of use of corticosteroids on survival.

Although our study was the first meta-analysis concerning NLR in PCa prognostication, there were several limitations need to be addressed. First, vast majority of included publications employed samples of Caucasian ethnicity, thus the evaluation of OS and RFS/PFS in Asians might be derived by chance because of sample insufficiency. Second, Shafique *et al.*^16^ recruited all types of PCa patients that could not be classified as any PCa types, which may contribute to heterogeneity when subgroup analysis and meta-regression were performed. Third, only publications in English were included, which could cause language bias for selection. Further investigations are needed to address the above-mentioned shortcomings.

In summary, our study demonstrated that elevated NLR predict poor OS and PFS/RFS in patients with PCa. Increased NLR showed consistent prognostic value in mCRPC patients and predict poor PFS/RFS in Asians, but not in Caucasians. The present findings could provide implications for clinical management of patients with PCa and further investigations involving large sample size and more ethnic backgrounds are needed.

## Methods

### Literature search

A comprehensive literature searching of Pubmed, Embase and Web of Science database was conducted. The search strategy included the combinations of the following key words: (NLR OR neutrophil-lymphocyte ratio OR neutrophil-to-lymphocyte ratio) AND (prostate cancer OR prostate carcinoma OR prostatic neoplasms OR PCa) AND (prognosis OR survival OR outcome OR recurrence). The last search was updated on October 17th, 2015. We also manually checked the reference list to identify additional publications. The published language was limited to English.

### Inclusion and exclusion criteria

Inclusion criteria for publication selection were as follows: (i) the diagnosis of PCa for patients was histopathologically confirmed; (ii) the value of NLR was obtained for blood sample testing; (iii) investigated the association of NLR with PFS, RFS or OS; (iv) provided HRs and 95% CIs for NLR in OS and (or) PFS/RFS, or HRs and 95%CIs could be calculated according to the raw data provided in the article; (v) defined the cut-off value of increased NLR; (vi) published in English language.

Major exclusion criteria were as follows: (i) letters, editorials, review articles; (ii) failed to provide data of interest or insufficient data to estimate HRs and 95%CIs; (iii) failed to identify the cut-off value for elevated NLR; (iv) animal studies and irrelevant studies.

### Data extraction

Two investigators (XB,G and XS,G) independently gathered information from each eligible study. The following data was extracted: surname of the first author, study country, year of publication, ethnic origin of the subjects, sample size, subtype of PCa, treatment method, cut-off value defining elevated NLR and HRs with corresponding 95% CIs for PFS/RFS and(or) OS. Discrepancies between the two investigators were settled by discussion.

### Statistical analysis

The HR and the corresponding 95%CI were used to assess the prognostic efficiency of NLR on PCa. HR and 95% CI were directly extracted from each single study, if provided, or calculated according to the methods clarified by Tierney^40^ *et al.* Cochran’s Q test and Higgins I-squared statistic were applied to test the heterogeneity of pooled data. I^2^ > 50% and p < 0.1 indicated significant heterogeneity and random-effects model was adopted to combine the effective value. I^2^ < 50% and p > 0.1 were considered as no heterogeneity and a fixed-effects model was then adopted. Sources of inter-study heterogeneity were explored using subgroup analysis and meta-regression. Begg’s funnel plot was used to evaluate publication bias. Sensitivity analyses were carried out to access the robustness of the results. All p values were two-tailed, and statistical significance level was set at p < 0.05. All statistical analyses were conducted using STATA 12.0 software (STATA Corporation, College Station, TX).

## Additional Information

**How to cite this article**: Gu, X. *et al.* Prognostic significance of neutrophil-to-lymphocyte ratio in prostate cancer: evidence from 16,266 patients. *Sci. Rep.* **6**, 22089; doi: 10.1038/srep22089 (2016).

## Figures and Tables

**Table 1:** Characteristics of the included studies.

Study	Year	Country	Ethnicity	Sample size	PCa type	Treatment method	Cut-off value Of NLR	Survival analysis
Shafique^16^	2012	UK	Caucasian	709	All PCa	NR	5	OS
Linton^15^	2013	Australia	Caucasian	182	mCRPC	Chemotherapy	5	OS
Nuhn^12^	2014	USA	Caucasian	247	mCRPC	Chemotherapy	3	OS
Sumbul^25^	2014	Turkey	Asian	33	mCRPC	Chemotherapy	3	PFS
Templeton^13^	2014	Canada	Caucasian	357	mCRPC	Chemotherapy	3	OS
Bahig^24^	2015	Canada	Caucasian	950	Localized PCa	Radiotherapy	3	OS,PFS
Kwon^23^	2015	USA	Caucasian	217	Localized PCa	Prostatectomy	2.6	RFS
Langsenlehner^14^	2015	Austria	Caucasian	415	Localized PCa	Radiotherapy	5	OS,PFS
Lee^22^	2015	Korea	Asian	1367	Localized PCa	Prostatectomy	2.5	RFS
Lorente^21^	2015	UK	Caucasian	755	mCRPC	Chemotherapy	3	OS,PFS
Poyet^20^	2015	Switzerland	Caucasian	399	Localized PCa	Prostatectomy	2.67	RFS
Sharma^19^	2015	USA	Caucasian	8350	Localized PCa	Prostatectomy	5	OS,PFS
Soest1^17^	2015	Netherland	Caucasian	1224	mCRPC	Chemotherapy	2	OS
Soest2^17^	2015	Netherland	Caucasian	1006	mCRPC	Chemotherapy	2.1	OS
Yao^18^	2015	Japan	Asian	55	mCRPC	Chemotherapy	3.5	OS,PFS

NR: not reported; OS: overall survival; PFS: progression-free survival.

**Table 2:** Summary of the subgroup analysis results of NLR on OS and PFS/RFS.

Outcome	Variable	No. of studies	No. of patients	Model	HR (95%CI)	Heterogeneity		Meta-regression P value
						Ph	I^2^(%)	
OS	All	11	14250	R	1.38(1.22–1.56)	0.002	64.1	
	PCa type							0.01
	mCRPC	7	3826	F	1.44(1.32–1.57)	0.238	25	
	Localized PCa	3	9715	R	1.16(0.98–1.36)	0.109	54.9	
	All PCa	1	709	—	1.55(0.97–2.47)	—	—	
	Sample size							0.19
	>400	7	13409	R	1.32(1.16–1.49)	0.005	67.8	
	<400	4	841	F	1.61(1.31–1.99)	0.252	26.6	
	NLR cut-off							0.756
	≤3	6	4539	R	1.34(1.24–1.45)	0.009	67.3	
	>3	5	9711	R	1.18(1.06–1.31)	0.066	54.6	
PFS/RFS	All	9	12541	R	1.24(1.05–1.46)	0.003	65.3	
	Ethnicity							
	Asian	3	1455	F	1.42(1.11–1.82)	0.345	6	
	Caucasian	6	11086	R	1.18(0.98–1.43)	0.003	72.3	
	PCa type							
	mCRPC	3	843	F	1.45(1.19–1.77)	0.37	0	
	Localized PCa	6	11698	R	1.16(0.97–1.39)	0.01	66.6	
	Sample size							
	>400	5	11837	R	1.28(1.00–1.65)	0.001	78.8	
	<400	4	704	F	1.14(0.98–1.33)	0.255	26.1	
	NLR cut-off							
	≤3	6	3721	F	1.17(1.06–1.29)	0.203	30.9	
	>3	3	8820	R	1.82(0.77–4.3)	0	87.3	

F: fixed-effects model; R: random-effects model.
